# Preliminary comparisons between a point-of-care ketometer and reference method using Steller sea lion pup whole blood and plasma

**DOI:** 10.1093/conphys/coad104

**Published:** 2024-01-27

**Authors:** Stephanie G Crawford, Robert H Coker, Lorrie D Rea

**Affiliations:** Department of Biology and Wildlife and Institute of Northern Engineering, University of Alaska Fairbanks, 1764 Tanana Loop, Fairbanks, Alaska 99775, USA; Montana Center for Work Physiology and Exercise Metabolism, University of Montana, 101 McGill Hall, 32 Campus Drive, Missoula, Montana 59812, USA; Institute of Northern Engineering, University of Alaska Fairbanks, 1764 Tanana Loop, Fairbanks, Alaska 99775, USA

**Keywords:** Fasting, ketometer, pinniped, rehabilitation

## Abstract

We evaluated the Precision Xtra™ ketometer as part of a larger study categorizing fasting status of free-ranging Steller sea lion (*Eumetopias jubatus*; SSL) pups which necessitated the identification of plasma β-hydroxybutyrate concentrations ([β-HBA]) around a threshold of <0.3 and ≥0.3 mmol/l. Whole blood samples mixed with sodium heparin (NaHep) or ethylenediaminetetraacetic acid liquid anticoagulants were tested <10 minutes after collection (*n* = 14; triplicate technical replicates). Plasma (stored at −80°C, NaHep, *Thaw1*) measured via our laboratory’s *Reference Assay* (Sigma Aldrich, St. Louis, MO, Kit #MAK041) served as the standard [β-HBA] for ketometer comparisons. Our observed β-HBA range (0.0–1.6 mmol/l), consistent with published [β-HBA] of free-ranging Otariid pups, represented the lower 20% of the ketometer’s range (0.0–8.0 mmol/l). The maximal coefficient of variation (%CV) of ketometer technical replicates was 9.1% (NaHep, whole blood). The majority of ketometer technical replicate sets (84%, including all matrices, anticoagulants and thawings) were identical (CV = 0%). We found linear relationships and agreement of ketometer [β-HBA] between whole blood preserved with different anticoagulants and between whole blood and plasma (*Thaw1*) measurements. The ketometer produced results with linearity to the *Reference Assay* for both whole blood and plasma (*Thaw1*). We identified a non-linear relationship between plasma at *Thaw1* and *Thaw2* (tested four months apart, NaHep), as only samples with higher SSL [β-HBA] decreased in concentration, and all others remained the same. With respect to categorizing SSL pup fasting in our larger study, the ketometer’s *%Accuracy*, %*Sensitivity* and %*Specificity* for samples with *Reference Assay* β-HBA <0.2 and >0.4 mmol/l were 100%. We adopted a modified procedure: plasma samples with mean ketometer concentrations ±0.1 mmol/l of 0.3 mmol/l β-HBA were re-evaluated using the *Reference Assay*, improving measurement precision from tenths (ketometer) to thousandths (assay) mmol/l. The Precision Xtra™ ketometer was valuable to our application over the range of [β-HBA] observed in SSL pup plasma and whole blood samples.

## Teaser

We demonstrated method agreement between *Reference Assay* concentrations of beta-hydroxybutyrate (#x03B2;-HBA, a ketone) and measurements obtained from freshly collected whole blood or thawed plasma via ketometer over the observed range (0.0#x2013;1.6#x00A0;mmol/l #x03B2;-HBA) in 14 young, free-ranging Steller sea lion pups.

## Introduction

Point-of-care ketometers, originally developed for human patients to self-monitor the condition of diabetic ketoacidosis, have in recent years routinely been used by veterinarians and dairy producers to monitor ketosis in domesticated dairy animals, such as cows, sheep and goats (Endecott *et al.,* 2004; Caldwell and Martineau, 2007; Burke *et al.,* 2008; Oetzel and McGuirk, 2008; Panousis *et al.,* 2012, 2017, 2018; Bach *et al.,* 2016; Ghanem *et al.,* 2016; Fiorentin *et al.,* 2017; Megahed *et al.,* 2017). We selected the Precision Xtra™ (Abbott Laboratories, Abbott Park, IL) ketometer to evaluate Steller sea lion pup (SSL, *Eumetopias jubatus*) β-hydroxybutyrate concentrations ([β-HBA]) in whole blood and plasma. Prior studies demonstrated the high accuracy of the Precision Xtra™ ketometer, showing agreement between the ketometer and laboratory results for β-HBA with sensitivity and specificity >90% (Caldwell and Martineau, 2007; Burke *et al.,* 2008; Oetzel and McGuirk, 2008). This ketometer’s utility has also been demonstrated in field research of wild birds (Lindholm *et al.,* 2018; Morales *et al.,* 2020), but we are unaware of a validated point-of-care ketometer for any mammalian wildlife species. Our primary objectives were to assess the Precision Xtra^TM^ ketometer’s (1) accuracy when testing thawed plasma samples, and (2) specifically to reliably allow us to identify plasma samples above and below a threshold of 0.3 mmol/l β-HBA, necessary for classifying the fasting category of free-ranging SSL pups (Crawford *et al.,* 2023).

### From the manufacturer

The Precision Xtra™ ketometer requires 1.5 μl of sample and displays a [β-HBA] between 0.0 and 8.0 mmol/l within 10 seconds. As the intended user of this meter is a diabetic human, it is designed to measure [β-HBA] in capillary whole blood; however, the package insert indicates that venous whole blood collected into sodium heparin (NaHep) or ethylenediaminetetraacetic acid (EDTA) blood collection tubes can be tested on this meter within 30 minutes of collection. Per the manufacturer, this meter was not designed for testing serum or plasma. It is calibrated by the manufacturer to plasma [β-HBA] obtained via the Randox assay kit (Randox Laboratories, Antrim, UK, Kit #RB1007). This meter also has a broad functional temperature range (0–30°C), making it ideal for many field settings.

The Precision Xtra™ utilizes a direct electrochemical test for analysis. When in contact with the test strip, the β-HBA within the sample produces an oxidation reaction with an enzyme on the test strip (hydroxybutyrate dehydrogenase). β-HBA is oxidized into acetoacetate, NAD^+^ is reduced to NADH and finally an electron transfer-mediated molecule re-oxidizes NADH to NAD^+^, resulting in a measurable electrical current. The current is proportional to the [β-HBA], and the ketometer displays the conversion in tenths of mmol/l. Depending upon the research question being evaluated, a ketometer or laboratory assay may be more appropriate (Table 1).

**Table 1:** Comparison of features of the two test methods, the *Reference Assay* and the Precision Xtra™ ketometer

	Sigma-Aldrich kit MAK-041Colorimetric *Reference Assay*	Precision Xtra™ ketometerPoint-of-care instrument
Tissue matrices	PlasmaSerumUrine	Capillary whole bloodVenous whole blood^a^Plasma^b^
Sample volume	30 μl/dilution + 20 μl for subsequent dilutions	1.5 μl/test strip
Time to results	~6 h	10 s
Measurement precision	0.000 mmol/l	0.0 mmol/l
Cost per sample^c^	~$35/triplicate technical replicates	~$6/triplicate technical replicates
Storage conditions Test materials Samples	−20°C−40 to −80°C	0 to +30°C0 to +30°C
Batching	Up to 15 samples can be run concurrently in triplicate	N/A
Shelf stability	Test materials <1 year when stored according to manufacturer	Test strips ~2 years
Other equipment and supplies needed	Plate readerPipetters and tips96-Well platesVortex	Ketometer ~$60

^a^Collected into blood tubes treated with NaHep and EDTA anticoagulants.

^b^Plasma not specified by manufacturer, but primary aim of this study is to assess the use of this tissue (NaHep anticoagulant).

^c^Costs in USD, 2016.

### Fasting in Steller sea lion pups

A young SSL pup remains on the natal rookery while the dam travels to sea to forage before returning to provision her pup (Milette and Trites, 2003; Maniscalco *et al.,* 2006; Burkanov *et al.,* 2011). Depending upon the duration of the dam’s absence, the pup may enter a fasting state, transitioning through three discrete phases distinguished by the predominant endogenous fuel supporting metabolic demands while maintaining homeostasis (Castellini and Rea, 1992). Storage products of carbohydrates (e.g. hepatic glycogen) and lipids (e.g. triacylglycerides) support metabolic demands during Phases I and II of fasting, respectively (Castellini and Rea, 1992). Transition into Phase III of fasting occurs once lipids have largely been exhausted; protein catabolism now supports demands. When endogenous resources can no longer support homeostasis, the pup progresses into a starvation state (Castellini and Rea, 1992). Maximal circulating ketone concentrations, therefore, occur during Phase II (lipid oxidation) through the transition into Phase III but would not be elevated (>0.3 mmol/l) during Phase I or Phase III of fasting or in a starvation state.

Fasting-adapted species, such as SSL, do not reach concentrations of circulating ketones, resulting in the condition of ketoacidosis. In prior studies of Otariid pups, the reported [β-HBA] were <1.5 mmol/l (Castellini *et al.,* 1993; Rea *et al.,* 1998, 2000; Arnould *et al.,* 2001), with one notable exception: concentrations approaching 4.0 mmol/l were documented in subantarctic fur seal pups (*Arctocephalus tropicalis*), which have a distinctly different life history than other Otariid species, resulting in pups fasting for exceedingly long durations in comparison with other related species (Guinet *et al.,* 2004; Verrier *et al.,* 2012). We do not anticipate measuring [β-HBA] in excess of 2.0 mmol/l, based on previous research for SSL (Castellini *et al.,* 1993, Rea *et al.*, 1998, 2000, 2009).

## Materials and Methods

The study animals for this initial ketometer evaluation were free-ranging SSL pups, <6 weeks of age, born on the Chiswell Island rookery in southcentral Alaska in 2016. These pups were handled as part of collaborative ongoing research by the Alaska SeaLife Center (ASLC) and the Alaska Department of Fish and Game (ADF&G) under Marine Mammal Protection Act permit numbers 18 537–00 and 18 438–00. Whole blood and plasma samples were provided to the University of Alaska Fairbanks (UAF) by the permitted agency for use in this study, only after fulfilling all other sample volume requirements for the ongoing and long-term research projects of ADF&G and ASLC. The sample size was determined by the conditions of the Federal handling permit, including guidelines to minimize the handling times of each individual pup and to expedite and minimize researcher disturbance to the rookery. The UAF Office of Research Integrity indicated that an additional Institutional Animal Care and Use protocol was not necessary, as this laboratory project utilized tissue samples collected for ADF&G and ASLC research projects; no co-authors were involved in the handling or sampling of live animals. Blood samples were drawn from the vein of the caudal gluteal plexus (Rea *et al.,* 1998; Keogh *et al.,* 2013) of anesthetized SSL pups (Heath *et al.,* 1997) directly into commercially-available blood collection tubes treated with NaHep and EDTA liquid anticoagulants and filled to manufacturer-specified volume for each blood tube.

The calibration of the ketometer was tested at the beginning of each day of use using low (0.4–0.8 mmol/l) and high (3.1–5.1 mmol/l) control solutions provided by the manufacturer. Within 10 minutes of collecting a blood sample, the whole blood [β-HBA] was measured by the Precision Xtra™ ketometer in triplicate technical replicates following manufacturer instructions for both types of anticoagulant blood collection tubes. Samples in blood collection tubes were kept chilled in the field until centrifuging at the end of the day. Hematocrit and total protein measurements were also made at the end of the field collection day. Plasma aliquots were frozen and stored at −80°C. Approximately nine months following the field collections, the [β-HBA] of thawed (*Thaw 1*) plasma aliquoted from NaHep-treated blood collection tubes was measured using both the Precision Xtra™ ketometer and the *Reference Assay*, the established method utilized in our research laboratory (Castellini and Costa, 1990; Castellini *et al.,* 1993), via a commercially-available assay kit (Sigma Aldrich, now Millipore Sigma, St. Louis, MO, Kit #MAK041). The [β-HBA] plasma (*Thaw 1*) data obtained via *Reference Assa*y for these 14 SSL pups were also utilized in Crawford *et al.*’s (2023) study that evaluated the fasting status of 1528 SSL pups.

Both the plasma *Reference Assay* and ketometer tests were conducted on the same day in triplicate technical replicates at *Thaw 1*. Data were reported as the mean (mmol/l) of the technical replicates, and the percent coefficient of variation (%CV) was calculated for each set of technical replicates; the desired %CV was <10% to meet laboratory standards. The remaining plasma samples were refrozen to −80°C. Four months later, these same plasma samples (*Thaw 2*) were again tested using only the Precision Xtra™ to evaluate the effect of an additional freeze–thaw cycle on the accuracy of the results. Intra-assay mean and median %CV were calculated for each unique sample type per method.

Results obtained from the Precision Xtra™ ketometer were compared with each other (e.g. whole blood and plasma both measured via ketometer) and to concentrations obtained via the *Reference Assay*. To compare measurements from these two methods, we used the non-parametric Passing–Bablok regression (Passing and Bablok, 1983; Bilić-Zulle, 2011) package in XLSTAT 2023 (Addinsoft, 2023). Other method evaluation techniques (e.g. Bland–Altman) were not available to us as our data violated test assumptions (Giavarina, 2015; Dogan, 2018; Taffé *et al.,* 2022).

To quantify this ketometer’s performance related to meeting our study’s specific needs, and under the assumption that the [β-HBA] values obtained from the *Reference Assay* were ‘true’, we calculated the percentage *Accuracy*, *Sensitivity* and *Specificity* for each matrix, anticoagulant and *Thaw* condition combination (Bartaloo *et al.,* 2015; Table 2). ‘True positives’ (TP) used in these calculations traditionally refer to an individual with the ‘disease’ being studied, confirmed by both test methods; some prior evaluations of the Precision Xtra™ on domesticated species considered the positive condition to be ketoacidosis for their assessment of the ketometer. As fasting-adapted species do not accumulate circulating ketone bodies in concentrations resulting in ketoacidosis, we defined a TP as a SSL pup sample with *Reference Assay* and ketometer [β–HBA] ≥0.3 mmol/l (i.e., SSL pups that are ‘positive’ for ketone production, documented by Rea *et al.* (2000) when fasting durations exceeded 16 hours). While the condition of fasting is not a ‘disease’, one of our objectives was to evaluate this ketometer’s performance in the identification of plasma samples above and below a threshold of 0.3 mmol/l β-HBA, essential to categorizing the fasting status of free-ranging SSL pups as part of our larger investigation (Crawford *et al.*, 2023).

**Table 2:** Description of ketometer test performance evaluations: the conditions required for classification as TP/TN and FP/FN are detailed in (a). Formulas (Bartaloo *et al.,* 2015) and descriptions for three evaluation metrics—percents *Accuracy*, *Sensitivity*, and *Specificity*—are outlined in (b).

(a)	Test Outcome	Abbreviation	Reference Assay [β-HBA]	Ketometer [β-HBA]
	True positives	TP	≥0.3 mmol/l	≥0.3 mmol/l
	True negatives	TN	<0.3 mmol/l	<0.3 mmol/l
	False positive	FP	≥0.3 mmol/l	<0.3 mmol/l
	False negative	FN	<0.3 mmol/l	≥0.3 mmol/l
**(b)**	**Metric**	**Performance** **E** **valuated**		**Formula**
	%Accuracy	TP & TN correctly identified with respect to overall sample size (*n*)		(TP + TN)/*n*
	%Sensitivity	Correctly identified TP		(TP)/(TP + FN)
	%Specificity	Correctly identified TN		(TN)/(TN + FP)

## Results

**Figure 1:**
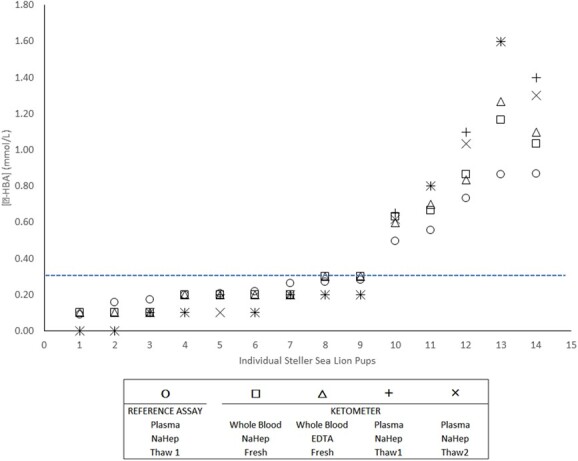
The mean [β-HBA] values obtained via *Reference Assay* are shown as open circles. The remaining mean [β-HBA] data were generated by the Precision Xtra™ ketometer for fresh whole blood (squares: NaHep, triangles: EDTA) and plasma (+: *Thaw 1*, ✕: *Thaw 2*, both NaHep). The ketometer underestimated [β-HBA] when *Reference Assay* measurements were <0.25 mmol/l, and conversely, the ketometer overestimated *Reference Assay* measurements were ≥0.25 mmol/l. The dashed blue line represents the metabolite threshold applied to categorize the fasting status of Steller sea lion pups (Crawford *et al*., 2023). The figure is sorted in ascending order by *Reference Assay* mean values.

Hematocrit and total protein measurements made at the end of the day of capture ranged between 31.0 and 44.0% (x- = 36%) and 6.0 and 6.8 g/dl (x- = 6.4 g/dl), respectively. The Precision Xtra™ ketometer passed all daily calibration tests using the low- and high-concentration controls. Tests were conducted within the instrument’s temperature range (per manufacturer) in both the field (12–19°C) and laboratory (20–22°C) settings. While the potential range of the Precision Xtra™ was 0.0–8.0 mmol/l β-HBA, observations from SSL pups in this study were all within the lower 20% of this range (0.0–1.6 mmol/l; Fig. 1); we were therefore unable to evaluate this meter’s performance over the upper 80% of its range.

The %CVs were <10% for all sets of technical replicates (for all matrices, anticoagulants and thaw cycles) run on the Precision Xtra™ ketometer (Table 3). The majority (84%) of ketometer technical replicates within a set were identical, resulting in a CV = 0%. Each set of ketometer technical replicates with CVs exceeding 0% were associated with *Reference Assay* ketone concentrations (0.495–0.871 mmol/l) in the higher range of concentrations observed in SSL. Whole blood collected into NaHep-treated blood collection tubes had the most technical replicate sets with CV > 0% (*n* = 4 of 14), as well as the maximal observed ketometer CV (9.1%; Table 3).

**Table 3:** Mean [β-HBA] from triplicate technical replicates (mmol/l) and respective %CV are presented for the measurements obtained via *Reference Assay* and ketometer for all combinations of matrices, anticoagulants and thaw regime (*n* = 14 Steller sea lion pups).

	**Technique**	Reference Assay	Ketometer			
	Matrix	Plasma	Plasma	Plasma	Whole blood	Whole blood
	Anticoagulant	NaHep	NaHep	NaHep	NaHep	EDTA
	Thaw	1st Thaw	1st Thaw	2nd Thaw	—	—
Individual pup number	1	0.091 9.6%	0.0 0.0%	0.0 0.0%	0.1 0.0%	0.1 0.0%
	2	0.158 10.2%	0.0 0.0%	0.0 0.0%	0.1 0.0%	0.1 0.0%
	3	0.173 6.2%	0.1 0.0%	0.1 0.0%	0.1 0.0%	0.1 0.0%
	4	0.198 6.6%	0.1 0.0%	0.1 0.0%	0.2 0.0%	0.2 0.0%
	5	0.206 10.6%	0.2 0.0%	0.1 0.0%	0.2 0.0%	0.2 0.0%
	6	0.219 1.8%	0.1 0.0%	0.1 0.0%	0.2 0.0%	0.2 0.0%
	7	0.263 2.3%	0.2 0.0%	0.2 0.0%	0.2 0.0%	0.2 0.0%
	8	0.271 4.0%	0.2 0.0%	0.2 0.0%	0.3 0.0%	0.3 0.0%
	9	0.282 6.1%	0.2 0.0%	0.2 0.0%	0.3 0.0%	0.3 0.0%
	10	0.495 3.6%	0.7 8.7%	0.7 8.7%	0.6 0.0%	0.6 0.0%
	11	0.556 5.5%	0.8 0.0%	0.8 0.0%	0.7 9.1%	0.7 0.0%
	12	0.733 8.9%	1.1 0.0%	1.0 5.6%	0.9 6.7%	0.8 6.9%
	13	0.867 2.3%	1.6 0.0%	1.6 0.0%	1.2 4.9%	1.3 4.6%
	14	0.870 2.2%	1.4 0.0%	1.3 0.0%	1.0 5.6%	1.1 0.0%
Mean [β-HBA]		0.384	0.475	0.454	0.433	0.443
Median [β-HBA]		0.267	0.20	0.20	0.25	0.25
Minimal %CV		1.9%	0.0%	0.0%	0.0%	0.0%
Maximal %CV		10.6%	8.7%	8.7%	9.1%	6.9%
Mean intra-assay %CV		5.7%	0.6%	1.0%	1.9%	0.8%
Median intra-assay %CV		5.8%	0.0%	0.0%	0.0%	0.0%

During our initial examination of the data (Fig. 1), we noted that the performance of the ketometer varied along the observed range of [β-HBA]. For both whole blood and plasma, the ketometer underestimated the [β-HBA] when the *Reference Assay* [β-HBA] results were <0.25 mmol/l; conversely, the ketometer method overestimated [β-HBA] compared with the *Reference Assay* when ketones were ≥0.25 mmol/l. The magnitude of the under- and over-estimation was greater for plasma than whole blood (Fig. 1).

**Figure 2:**
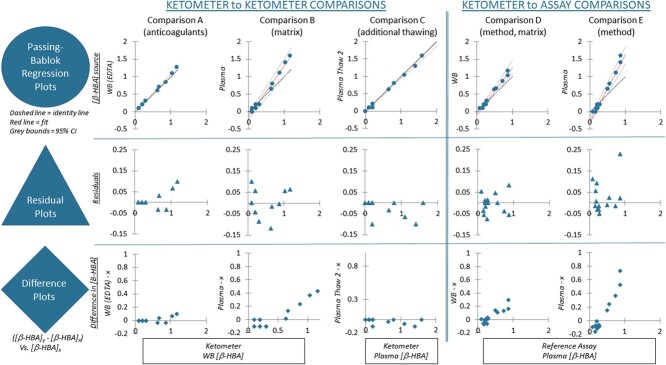
Visualization of results of the Passing–Bablok regression for each of the five comparisons (A–E, outlined in Table 4; *n* = 14 Steller sea lion pups). The first row of plots (datapoints are filled circles) are the Passing–Bablok regression plots: the dashed line is the identity line, the red line is the fitted line and the grey bounds indicate the 95% confidence intervals (CI) around the fitted line. The second row of plots (datapoints are triangles) are the residual plots from these analyses. The final row of plots (datapoints are diamonds) are difference plots demonstrating the deviation of the *x* [β-HBA] from the *y* [β-HBA] at the different concentrations of *x*. The *x*-axis labels are found in the boxes along the bottom of the figure. All references to whole blood (WB) refer to samples with the anticoagulant NaHep, unless otherwise specified. Similarly, all references to plasma refer to samples at *Thaw 1* unless otherwise specified. Only one comparison—*Thaw 1* plasma versus *Thaw 2* plasma—was found to have a non-linear relationship (comparison C, *P* < 0.001). The fitted line of comparison C has tight CIs; however, the residual plots and difference plots indicate that the deviations are not evenly distributed around the fitted line.

In comparison A of our Passing–Bablok regression analyses (Table 4; Fig. 2), we found the field ketometer [β-HBA] of whole blood collected into EDTA- and NaHep-treated blood tubes to be highly linearly correlated (H = 3.037, *P* = 0.660); no variability attributable to anticoagulant was found. Although the manufacturer specified that the Precision Xtra™ ketometer was not intended for use with plasma or serum, we also found that the ketometer’s [β-HBA] measurements on freshly collected whole blood and plasma (*Thaw 1*) (both from NaHep-treated blood collection tubes) were linearly correlated (H = 5.082, *P* = 0.541, comparison B, Table 4, Fig. 2).

**Table 4:** Five comparisons (A–E) are outlined. Results from a Passing–Bablok regression conducted for each comparison are presented. Significant *P*-values from the two-tailed linearity test are highlighted by ***bold italics***, evaluating the H_0_ = the relationship between the two variables is linear.

**Ketometer (*y*) to ketometer (*x*)**						
	**Comparison by**	**Dependent (** ***y*** **)**	**Independent (** ***x*** **)**	**Test statistic (H)**	***P*** **-value (α = 0.05)**	**Passing–Bablok regression equation**
A	Anticoagulant^a^	Whole blood^b^	Whole blood	3.037	0.660	*y* = 1*x* + 0.0
B	Matrix	Whole blood	Plasma^c^	5.082	0.541	*y* = 1.440*x* − 0.146
C	Additional thawing	Plasma	Plasma^d^	2.716	***<0.001***	*y* = 1*x* + 0.0
**Ketometer (** ***y*** **) to Reference Assay (** ***x*** **)**						
	**Comparison by**	**Dependent (** ***y*** **)**	**Independent (** ***x*** **)**	**Test statistic (H)**	***P*** **-value (α = 0.05)**	**Passing–Bablok regression equation**
D	Method	Plasma	Whole blood	5.082	1.000	*y* = 1.337*x* − 0.077
E	Method	Plasma	Plasma	5.082	0.938	*y* = 1.912*x* − 0.287

^a^All samples in table were aliquoted from blood collection tubes containing NaHep anticoagulant unless otherwise specified.

^b^EDTA anticoagulant.

^c^Plasma throughout table refers to *Thaw 1* unless otherwise specified.

^d^ *Thaw 2.*

A non-linear relationship was found between plasma [β-HBA] measured by ketometer at *Thaw 1* and *Thaw 2* (H = 2.716, *P* < 0.001, comparison C, Table 4, Fig. 2). While 10 of 14 samples showed no change between thawings, the four other samples decreased in mean [β-HBA] by 0.03–0.10 mmol/l following *Thaw 2*, resulting in 10 residuals lying on the fitted line and the other four below (Fig. 2). Similarly, the difference in measurements for comparison C are all ≤0, resulting in an uneven distribution around the zero line. For the purposes of our larger study (Crawford *et al.*, 2023), our classification of the SSL pup fasting phase would not have differed as a result of any of the changes observed in [β-HBA] following *Thaw 2.* Comparisons D and E between ketometer and *Reference Assay* [β-HBA] (plasma, *Thaw 1*) were both found to be linearly correlated: whole blood (H = 5.082, *P* = 1.000) and plasma (H = 5.082, *P* = 0.938), respectively (Table 4, Fig. 2).

For the computation of the *%Accuracy, %Sensitivity*, and *%Specificity*, we first needed to determine the distribution of TP (β-HBA ≥0.3 mmol/L) and true negatives (TN) for the condition of ketone production (see Table 2 for definitions and equations). Using *Reference Assay* [β-HBA] of plasma at *Thaw 1,* we identified nine TP and five TN (*n* = 14). The *%Sensitivity* of the Precision Xtra™ ketometer was 100% for all sample type/conditions evaluated (Table 5), as were the %*Accuracy* and %*Specificity* for plasma measured at both *Thaw 1* and *Thaw 2* (Table 5). Two individual SSL pup whole blood samples resulted in false positives (FP) for both anticoagulant types, reducing the %*Accuracy* and %*Specificity* to 85.7 and 71.4%, respectively, for both whole blood ketometer evaluations (Table 5). Both FP had ketometer measurements of 0.3 mmol/l (= the fasting classification threshold), while *Reference Assay* concentrations were 0.271 and 0.282 mmol/l.

## Discussion

Megahed *et al.* (2017) investigated the effect of sample temperature on the Precision Xtra™ results across different known [β-HBA]. While Megahed *et al.* (2017) did identify a significant effect of sample temperature, this was only for samples with [β-HBA] >3.0 mmol/l. While we did not evaluate sample temperature prior to testing, our ambient temperature ranged between 12 and 22°C, and all of our *Reference Assay* [β-HBA] were below the concentration where the temperature was observed to affect the results (maximal [β-HBA] = 1.6 mmol/l).

Megahed *et al.* (2017) also examined the effect of hematocrit on the Precision Xtra™ results across different known [β-HBA]. As this meter was designed for use on human blood samples, the authors point out that an assumption that their study animal (dairy cattle) has similar hematocrit levels as humans is made. The Precision Xtra™ manual indicates that the meter is for samples with hematocrit of 30–60%. All our observations from the SSL pups in our study fall within these thresholds. Additionally, all SSL hematocrit and plasma total protein measurements fell within the normal reference ranges established for SSL pups (32.2–47.1% and 5.4–7.0 g/dl, respectively; Lander *et al.,* 2013) with one exception: one male pup had a hematocrit of 31%.

We acknowledge that our investigation is limited by the small sample size and the phenomena that SSL and other fasting-adapted species do not concentrate [β-HBA], preventing examination of the full range capacity of this meter. While we applied the non-parametric Passing–Bablok regression, others have suggested a minimal sample size of 50 is ideal for this analysis, as small sample sizes produce wider confidence intervals, which increases the likelihood that these intervals include 0 for the intercept and 1 for the slope, which ultimately leads to a biased conclusion of method agreement (Passing and Bablok, 1985; Ludbrook, 2010). As we were only evaluating a small portion of the ketometer’s range (0.0–1.6 mmol/l), there is an increased probability of false-positive results (Passing and Bablok, 1985).

**Table 5:** Results of calculations of three ketometer performance evaluation metrics

	Sample description			
Metric	Plasma, *Thaw 1* NaHep	Plasma, *Thaw 2* NaHep	Whole blood, fresh NaHep	Whole blood, fresh EDTA
%*Accuracy*	100%	100%	85.71%	85.71%
%*Sensitivity*	100%	100%	100%	100%
%*Specificity*	100%	100%	71.43%	71.43%

See Table 2 for descriptions and formulas.

## Conclusions

We found the Precision Xtra™ ketometer to be a simple, accurate and cost-effective preliminary analysis tool for the range of measurements used to classify the fasting phases of SSL pups. We established that this ketometer can measure [β-HBA] in thawed plasma samples, meeting our initial study objective. We further demonstrated that for the majority of plasma [β-HBA] observed in SSL pups, this ketometer can accurately aid in our discrimination of samples <0.3 and ≥0.3 mmol/l, with one modification. Samples with a ketometer result between 0.2 and 0.4 mmol/l will be further analysed via the *Reference Assay* method, which has greater measurement precision. This procedural change will minimize the uncertainty around the fasting classification threshold (0.3 mmol/l). Therefore, the ketometer measurements we accept without re-evaluation by *Reference Assay* will all be associated with the range of [β-HBA] scoring 100% in all performance evaluation metrics (for both whole blood and plasma). If we had applied this analysis strategy to all samples used in our larger study, only 21% would have required further evaluation via the more costly, less shelf-stable *Reference Assay* kits. Further, this sample-filtering approach would have resulted in ~73% cost reduction for the β-HBA component of our broader research project (cost estimates calculated from 2016 pricing; Table 1).

We also suggest that this ketometer could become a valuable pup-side tool for those rehabilitating orphaned pinniped pups. Pups arriving at a rehabilitation facility in severely poor body condition (often classified as starvelings or as emaciated) and with [β-HBA] near or equal to 0.0 mmol/l have likely transitioned into Phase III of fasting or possibly into a starvation state. Re-feeding syndrome is more common in these scenarios (Winn, 2006; Fravel *et al.*, 2016; McKnight *et al.,* 2019; McRuer and Ingraham, 2019). While certainly a more rigorous evaluation of this ketometer would be necessary for application in a clinical setting with individual patients, with additional validation measures, rehabilitation facilities could use this as a tool to further inform their triage efforts and aid in the development of re-feeding plans.
